# Macroalgal Endophytes from the Atlantic Coast of Canada: A Potential Source of Antibiotic Natural Products?

**DOI:** 10.3390/microorganisms1010175

**Published:** 2013-12-12

**Authors:** Andrew J. Flewelling, Katelyn T. Ellsworth, Joseph Sanford, Erica Forward, John A. Johnson, Christopher A. Gray

**Affiliations:** 1 Department of Biology, University of New Brunswick, 100 Tucker Park Road, Saint John, NB E2L 4L5, Canada; E-Mails: d17x5@unb.ca (A.J.F.); katie.ellsworth@dal.ca (K.T.E.); m4skb@unb.ca (J.S.); erica.forward@unb.ca (E.F.); jaj@unb.ca (J.A.J.); 2 Department of Chemistry, University of New Brunswick, 100 Tucker Park Road, Saint John, NB E2L 4L5, Canada

**Keywords:** marine macroalgae, endophyte, antibacterial, antifungal

## Abstract

As the need for new and more effective antibiotics increases, untapped sources of biodiversity are being explored in an effort to provide lead structures for drug discovery. Endophytic fungi from marine macroalgae have been identified as a potential source of biologically active natural products, although data to support this is limited. To assess the antibiotic potential of temperate macroalgal endophytes we isolated endophytic fungi from algae collected in the Bay of Fundy, Canada and screened fungal extracts for the presence of antimicrobial compounds. A total of 79 endophytes were isolated from 7 species of red, 4 species of brown, and 3 species of green algae. Twenty of the endophytes were identified to the genus or species level, with the remaining isolates designated codes according to their morphology. Bioactivity screening assays performed on extracts of the fermentation broths and mycelia of the isolates revealed that 43 endophytes exhibited antibacterial activity, with 32 displaying antifungal activity. Endophytic fungi from Bay of Fundy macroalgae therefore represent a significant source of antibiotic natural products and warrant further detailed investigation.

## 1. Introduction

The threat to human health as a result of the growing emergence of microbial resistance to antibiotic agents is both real and significant; current forecasts predict that broad-scale antimicrobial ineffectiveness is imminent and we may soon once again face the problems that challenged medicine in the “pre-antibiotic” era [1,2,3]. There is, therefore, an urgent need to accelerate the discovery of antibiotic molecules to facilitate the development of new therapeutic agents with novel modes-of-action to combat infectious disease [4,5]. Endophytes of macroalgae have recently gained attention as an untapped source of biodiversity with potential to yield novel bioactive metabolites [6,7,8] and high proportions of isolates from both tropical [9] and temperate [10,11] environments have exhibited significant antibiotic activities. However, data relating to the bioactivity of endophyte assemblages obtained from a given algal species is limited; evaluating the true potential of macroalgal endophytes as a source of antibiotic compounds is therefore problematic. Recent reports also suggest that there may be significant differences in the endophyte assemblages found within tropical and temperate marine macroalgal hosts [9,11], although further work is required to confirm these preliminary observations. The objectives of this study were to perform a preliminary investigation of the endophytes from macroalgae from the Atlantic coast of Canada and evaluate their potential for the production of antimicrobial compounds.

## 2. Experimental Section

### 2.1. Algal Collection

Fourteen species of algae were collected from L’Etete, New Brunswick, Canada (45° 02.372′ N, 66° 53.424′ W) from August to November 2009. Seven species of red algae (*Porphyra umbilicalis*, *Porphyra purpurea*, *Palmaria palmata*, *Chondrus crispus*, *Devaleraea ramentacea*, *Mastocarpus stellatus* and *Polysiphonia lanosa*), four species of brown algae (*Fucus spiralis*, *Fucus vesiculosus*, *Saccharina* *latissima* and *Ascophyllum nodosum*) and three species of green algae (*Spongomorpha arcta*, *Ulva intestinalis* and *Ulva lactuca*) were investigated for the presence of endophytic fungi. In all cases algae with no obvious signs or symptoms of disease were used.

### 2.2. Surface Sterilization of Algae and Culture Techniques

The surfaces of the algal samples were sterilized by immersion in various sterilant solutions [11]. Prior to the isolation of endophytic fungi from marine algae, an optimal surface sterilization method was developed for each algal species (Table 1). Portions (5 cm^2^) of algal tissue were individually surface sterilized using the appropriate optimized technique, blotted dry on autoclaved paper towel and rubbed across the surface of 2 plates of 2% malt extract agar (MEA, Becton Dickinson, Sparks, MD, USA) prepared with artificial seawater (MEA-SW, 24.4 g·L^−1^; Instant Ocean^®^ Sea Salt, Cincinnati, OH, USA) to verify surface sterilization had been effective. Sterilized algal species were cut using a sterile cork borer and placed on Petri plates of 2% MEA and 2% MEA with seawater. Petri plates were sealed with Parafilm™ (Pechiney Plastic Packaging Company, Chicago, IL, USA) prior to incubation.

**Table 1:** Surface sterilization techniques used on marine algae collected from the Bay of Fundy, Canada. ^1^

Algal Species	Sterilant Immersion Duration (Seconds)	
	Bleach (5.25%)	Ethanol (70%)
*Chondrus crispus*	5	10
*Devaleraea ramentacea*	5	10
*Mastocarpus stellatus*	5	15
*Palmaria palmata*	0	15
*Polysiphonia lanosa*	0	10
*Porphyra purpurea*	0	10
*Porphyra umbilicalis*	0	10
*Ascophyllum nodosum*	0	15
*Fucus spiralis*	0	15
*Fucus vesiculosus*	0	15
*Saccharina latissima*	5	15
*Spongomorpha arcta*	0	20
*Ulva intestinalis*	0	20
*Ulva lactuca*	0	20

^1^ All surface sterilizations were confirmed by the absence of microbial colony formation on 2 surface verification plates (2% malt extract with artificial seawater).

### 2.3. Isolation of Endophytes

Petri plates containing surface sterilized macroalgal pieces were incubated for 14 days at room temperature (approximately 25 °C) under ambient light conditions and monitored daily for the presence of hyphae growing from the cut edges of the algal segments. The isolation frequency (IF) of emerging hyphae was determined for each species of algae collected [12]:

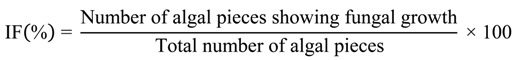
(1)
Endophytes growing from the cut edges of the segments were subcultured onto fresh media (2% MEA) to obtain pure isolates. Pure isolates were also grown on Czapek, potato dextrose, cornmeal and marine agars (Becton Dickinson, Sparks, MD, USA) to induce sporulation and differentiate between colony morphologies. Individual isolates from each algal species were then sorted into groups of homogeneous morphotypes, and a representative colony of each distinct isolate was used for the fungal identification, fermentation, and extraction.

### 2.4. Identification of Fungi

Fungal isolates were identified taxonomically through examination of colony and spore morphology with taxonomic classifications being confirmed by comparison of the internal transcribed spacer and 5.8S rRNA gene (ITS) DNA regions [13] with corresponding sequences available in the GenBank database (National Center for Biotechnology Information, U.S. National Library of Medicine, Bethesda, MD, USA).

The genomic DNA of all distinct fungal isolates was extracted using a DNeasy^®^ plant mini kit (Qiagen, Toronto, Ontario, Canada) and amplified by PCR as previously described [11]. Samples of amplified ITS DNA were submitted for sequencing (Génome-Québec; Montreal, Québec, Canada) along with the ITS 1 and ITS 4 primers. The sequences obtained were checked for ambiguity and submitted to the GenBank database and compared with existing GenBank sequence data using BLAST. In cases where electrophoresis indicated that the DNA extraction/ PCR procedure had been unsuccessful, the procedure was repeated on a fresh sample of the fungal isolate. 

Isolates identified taxonomically to species level had ≥99% sequence similarities to entries for conspecifics in GenBank, isolates identified to the genus level had ≥96% sequence similarities with congeneric species, and isolates identified to class level had sequence similarities ≥82% to entries from the corresponding taxonomic rank. Isolates that could not be unequivocally identified to species level based on morphological observations were only classified to the corresponding genus even when the sequence similarities ≥98% were obtained with sequence data for congeneric species in GenBank. If DNA from the ITS region was not isolated after three extraction/amplification attempts, the corresponding isolate was identified on morphological observations alone. Sterile isolates that did not provide sequence data upon repeated attempts were given codes according to their morphology in plate culture [9,14,15]. All distinct fungal isolates have been archived in the UNB Saint John fungal repository (Saint John, New Brunswick, Canada).

### 2.5. Preparation of Extracts

For each fungal isolate, a portion (5 mm^2^) of the fungal colony on solid medium was transferred to a 250 mL Erlenmeyer flask containing 2% Bacto™ malt extract broth (100 mL). Flasks were shaken (150 rpm) at room temperature under ambient light conditions for two weeks.

After incubation, the fungal mycelia from each culture were separated from the spent culture broth using vacuum filtration. Mycelia were extracted once with methanol (50 mL; Fisher Scientific, Ottawa, Ontario, Canada) in the dark for 24 h at 4 °C, solid residue and cell debris were removed by vacuum filtration, and the resulting solution was concentrated *in vacuo* to give a crude fungal extract. The spent culture broths were extracted 3 times with 50 mL ethyl acetate (Fisher Scientific) and the combined organic extracts were concentrated *in vacuo* to give a crude extract of the growth media. All crude extracts were stored at −20 °C until required.

### 2.6. Antibacterial and Antifungal Activity Assay

Antibacterial and antifungal activity against *Pseudomonas aeruginosa* (ATCC 10145), *Staphylococcus aureus* (ATCC 29213) and *Candida albicans* (ATCC 14053) was evaluated using a microbroth dilution antimicrobial susceptibility assay as previously described [11] with extracts being tested at a concentration of 200 μg·mL^−1^.

### 2.7. Statistical Analyses

Antimicrobial activities of extracts were compared to the negative control using the Kruskal-Wallis non-parametric test as the data was not normally distributed (Shapiro-Wilk, *p* < 0.05) and the variances were not equal (Levene’s test, *p* < 0.05). *Post hoc* analysis (stepwise-stepdown) was performed to determine which extracts differed from the negative control. Crude extracts were defined as biologically active if their effect on the growth of the test organism was significantly different (*p* < 0.05) to the negative control (0% inhibition). All statistical tests were performed using SPSS (PASW Statistics 18, IBM Corporation, Armonk, NY, USA).

## 3. Results and Discussion

### 3.1. Isolation of Endophytic Fungi from Marine Algae

The Bay of Fundy, with its extreme tidal range, presents a unique temperate habitat and supports a high diversity of marine macroalgal species [16,17], many of which have not been investigated for the presence of endophytes. Our results indicate that these algae support a sizeable and diverse assemblage of endophytes: fungi were isolated from 632 out of a total 3081 algal pieces resulting in an overall isolation frequency of 26% (Table 2). The isolation frequencies of endophytic fungi varied by host, with the highest isolation frequencies obtained from the red algae *P. palmata* and *P. umbilicalis*, at rates of 87% and 72% respectively (Table 2). The lowest isolation frequencies were from the red alga *C. crispus* at a rate of 0.08% (Table 2).

**Table 2:** Isolation frequencies and distinct number of isolates of endophytic fungi from marine algae collected from the Bay of Fundy, Canada.

Seaweed Species	Total Algae Segments	Pieces with Endophytic Growth	Isolation Frequency (%)	Distinct Fungal Taxa
*Chondrus crispus*	246	2	0.08	2
*Devaleraea ramentacea*	108	46	43	13
*Mastocarpus stellatus*	98	32	33	9
*Palmaria palmata*	167	146	87	7
*Polysiphonia lanosa*	67	12	18	5
*Porphyra purpurea*	85	17	20	2
*Porphyra umbilicalis*	217	156	72	3
*Ascophyllum nodosum*	152	16	11	1
*Fucus spiralis*	192	16	8	4
*Fucus vesiculosus*	109	6	6	1
*Saccharina latissima*	1138	95	8	13
*Spongomorpha arcta*	184	49	27	11
*Ulva intestinalis*	117	24	21	5
*Ulva lactuca*	201	15	8	3
Total	3081	632	26	79

Seventy-nine distinct endophyte species were isolated from the ten algal hosts (Table 3). The number of distinct fungal isolates obtained varied by host with a maximum of 13 endophytic fungal species from *D. ramentacea* and *S. latissima* to a minimum of one from *A. nodosum* and *F. vesiculosus* (Table 3). *Penicillium* spp. were isolated from seven algae: *C. crispus*, *D. ramentacea*, *M. stellatus*, *P. palmata*, *S. latissima*, *S. arcta* and *U. lactuca* with a total of 11 isolates representing the six species isolated (Table 3). Six distinct *Aspergillus* spp. were isolated from five algae species: *C. crispus*, *P. umbilicalis*, *A. nodosum*, *S. latissima* and *U. intestinalis* (Table 3). *Botrytis* sp. was isolated from two algal hosts (*D. ramentacea* and *P. palmata*; Table 3) as was *Aureobasidium pullulans* (*D. ramentacea* and *P. lanosa*; Table 3). *Cladosporium* sp., *Trametes versicolor*, *Coniothyrium* sp., Coelomycete I, *Hypoxylon* sp., *Helicomyces* sp. and *Botryotinia fuckeliana* were only isolated from one host (Table 3).

**Table 3:** Identification of endophytic fungi isolated from marine algae collected from the Bay of Fundy.

Host species	Isolate	Species	Accession number
*Chondrus crispus*	AF8-053	*Aspergillus* sp. I	KF572134
	AF8-129	*Penicillium crustosum* I	KF572146
*Devaleraea ramentacea*	AF8-079	*Cladosporium sp.*	KF572136
	AF8-081	Septate Pigmented I	-
	AF8-083	Septate Pigmented II	-
	AF8-085	Sterile Hyaline I	-
	AF8-087	Sterile Hyaline II	-
	AF8-089	*Botrytis* sp. I	-
	AF8-091	*Penicillium decumbens* I	KF572138
	AF8-093	Septate Pigmented III	-
	AF8-095	*Aureobasidium pullulans* I	-
*Devaleraea ramentacea*	AF8-109	*Trametes versicolor*	KF572133
	AF8-111	White Fluffy II	-
	AF8-113	*Coniothyrium sp.*	KF572141
	AF8-115	*Botrytis* sp. II	-
*Mastocarpus stellatus*	AF8-055	*Penicillium sp.*	-
	AF8-057	Red Yeast I	-
	AF8-059	Septate Pigmented IV	-
	AF8-061	Red Yeast II	-
	AF8-063	Sterile Hyaline III	-
	AF8-065	Sterile Hyaline IV	-
	AF8-067	Coelomycete I	-
	AF8-101	*Penicillium decumbens* II	KF572139
*Palmaria palmata*	AF8-069	Sterile Hyaline V	-
	AF8-071	*Hypoxylon sp.*	KF572135
	AF8-073	*Penicillium decumbens* III	KF572149
	AF8-075	*Penicillium chrysogenum* I	KF572137
	AF8-077	*Helicomyces* sp.	-
	AF8-103	*Penicillium crustosum* II	KF572147
	AF8-105	*Botrytis* sp. III	-
	AF8-107	Sterile Hyaline VI	-
	AF8-123	Sterile Hyaline VII	-
*Polysiphonia lanosa*	AF8-097	Sterile Hyaline VIII	-
	AF8-099	*Aureobasidium pullulans* II	KF572140
	AF8-117	Sterile Hyaline IX	-
	AF8-119	*Botryotinia fuckeliana*	KF572148
	AF8-121	Sterile Hyaline X	-
*Porphyra purpurea*	AF8-125	Sterile Hyaline XI	-
	AF8-127	Sterile Hyaline XII	-
*Porphyra umbilicalis*	AF8-049	Sterile Hyaline XIII	-
	AF8-051	Sterile Hyaline XIV	-
	AF8-131	*Aspergillus sydowii*	KF572145
*Ascophyllum nodosum*	AF1-141B	*Aspergillus sp*. II	-
*Fucus spiralis*	AF1-021A	Black Hyaline I	-
	AF1-021B	Septate Pigmented V	-
	AF1-021D	Pigmented Hyaline I	-
	AF1-021G	Sterile Hyaline XV	-
*Fucus vesiculosus*	AF2-059A	Sterile Hyaline XXI	-
*Saccharina latissima*	AF1-029A2	White Hyaline IV	-
	AF1-029B2	Black Hyaline II	-
	AF1-073C	*Penicillium chrysogenum* II	KF572144
	AF1-073D	*Aspergillus* sp. III	-
	AF1-073E	White Hyaline I	-
	AF1-073G	Sterile Beige I	-
	AF1-073N	Sterile Beige II	-
	AF2-055B	*Penicillium soppii* I	KF572151
	AF2-055F	Sterile Hyaline XVI	-
	AF2-055G	White Hyaline II	-
	AF2-055O	*Penicillium chrysogenum* III	KF572150
	AF2-055P	Pigmented Hyaline IV(Red)	-
*Spongomorpha arcta*	AF1-037C2	Sterile Hyaline XVII	-
	AF1-037F	Sterile Beige III	-
	AF1-153A	Pigmented Hyaline VII	-
	AF1-153B	Sterile Hyaline XVIII	-
	AF1-153C	White Hyaline III	-
	AF1-153D	*Penicillium spinulosum*	KF572143
	AF1-153H	Black Hyaline III	-
	AF1-153J	Pigmented Hyaline VI	-
	AF1-153L	Pigmented Hyaline V	-
	AF1-153M	*Penicillium* sp.	KF572152
	AF1-153N	*Penicillium soppii* II	-
*Ulva intestinalis*	AF2-063C	Sterile Hyaline XIX	-
	AF2-063E	Septate Pigmented VIII	-
	AF2-063F	Pigmented Hyaline VIII	-
	AF2-063G	*Aspergillus* sp. V	-
	AF2-063H	Septate Pigmented VI	-
*Ulva lactuca*	AF1-033A2	Septate Pigmented VII	-
	AF1-033B	Sterile Hyaline XX	-
	AF1-033C	*Penicillium chrysogenum* IV	KF572142

The majority of isolates (74%), however, could neither be identified morphologically nor through the use of molecular genetic techniques. These isolates were designated codes according to their plate morphology (Table 3). This proportion of sterile mycelia (74%) is high in comparison to other marine macroalgal endophytes from the North Atlantic (45%) [11] and may have contributed to a correspondingly lower success rate observed for the molecular identification of isolates in this study (26% compared with 55% for endophytes isolated from macroalgae of the Shetland Islands, UK) [11]. The magnitude of these discrepancies is surprising and may be due to the particular assemblage of fungal species isolated from the Bay of Fundy algae. Further work will be required to optimize procedures used for molecular identification of sterile algal endophytes in an effort to increase our ability to identify fungi isolated from this source.

*Penicillium* spp. and *Aspergillus* spp. are common endophytes of marine macroalgae [9,11], and represent nearly one quarter (24%, 19/79 isolates) of the distinct isolates obtained from macroalgae of the Bay of Fundy. The majority of fungi obtained from Bay of Fundy macroalgae were isolated as sterile mycelia and several of the endophytes, *i.e.*, *Botrytis* spp., *Hypoxylon* sp., and *Helicomyces* sp., have not been previously isolated from marine algal hosts. Many of the macroalgae investigated in this study, *S. latissima*, *S. arcta*, *P. purpurea*, *P. umbilicalis*, *P. palmata*, *M. stellatus* and *D. ramentacea*, have not been previously investigated for their endophytic fungi. Endophytes have previously been reported from *A. nodosum*, *F. spiralis*, *F. vesiculosus*, *P. lanosa*, *U. intestinalis* and *U. lactuca* [9,11,18,19,20,21,22,23,24,25,26,27,28,29,30]. However, there was a high degree of variability in the diversity of fungi obtained in these studies, which suggests limited host specificity of these fungal endophytes to their hosts.

A point of particular interest is the fact that none of the fungi isolated from the brown alga *A. nodosum* were identified as *Mycosphaerella ascophylli*, despite it being a well documented endophyte of that host [18,19,20,21,22,24,27,28,30]. Whilst it is possible that *M. ascophylli* may not have been isolated due to the particular sterilization and culture conditions used or the low growth rate of the fungus [19], it is more probable that *M. ascophylli* is represented in the *mycelia sterilia* obtained from *A. nodosum* as it is known to display a sterile morphotype of fine white septate hyphae [19,31].

### 3.2. Antimicrobial Screening Results of Algal-Derived Fungal Endophytes

Crude extracts of the endophyte isolates were screened against three pathogenic microorganisms, the Gram positive bacterium *Staphylococcus aureus*, the Gram negative bacterium *Pseudomonas aeruginosa* and the fungus *Candida albicans*. Extracts were defined as bioactive if they inhibited growth of the test organism in comparison to the negative control as indicated by heterogeneous subsets (*p* < 0.05) identified through *post hoc* testing of Kruskal-Wallis analyses. Seventy-eight crude extracts, tested at 200.0 μg·mL^−1^, inhibited at least one of the test microorganisms. Forty-four crude extracts significantly inhibited the growth of *S. aureus*, whereas, 18 and 36 extracts inhibited the growth of *P. aeruginosa* and *C. albicans*, respectively. These extracts were derived from 57 individual fungal isolates (Table 4) and comprised 39 mycelia-derived and 39 medium-derived crude extracts. Of the 156 crude extracts tested, 59 showed activity against only 1 test microorganism, 18 inhibited 2 microorganisms, and 1 showed activity against all 3 test microorganisms (Table 4). In this study, 73% of the isolates (57/78) were found to have antimicrobial activity against *P. aeruginosa*, *S. aureus*, and *C. albicans* (43/78, 55% antibacterial; 32/78, 41%, antifungal). The antimicrobial screening results of this study are comparable to those obtained from marine algae of the Shetland Islands, UK where 61% of the isolates (39/64) had activity against at least one of the same three test organisms (36/64 isolates antibacterial and 24/64 isolates antifungal) [11]. However, these antimicrobial screening results are in contrast to those obtained from endophytic fungi of macroalgae of the Southern Indian coast (82%, 31/38 isolates) [9] and the coasts of the North and Baltic Seas (83%, 249/300) [10]. The crude extracts showing strong antimicrobial bioactivity in this work should be investigated to identify the biologically active constituents responsible for the observed bioactivity.

**Table 4:** Activity of crude extracts showing significant inhibition to *S. aureus*, *P. aeruginosa* and *C. albicans*, obtained from endophytic fungi isolated from marine algae collected from the Bay of Fundy.

Fungal Taxa	Host Alga	Extract ^1^ Source	Inhibition (%) ^2^		
			SA ^3^	PA ^3^	CA ^3^
*Aspergillus* sp. I	*C. crispus*	Mycelia	-	18 ± 1	-
*Aspergillus* sp. I	*C. crispus*	Media	28 ± 3	-	15 ± 3
*Penicillium crustosum* I	*C. crispus*	Media	43 ± 1	-	-
*Cladosporium sp.*	*D. ramentacea*	Mycelia	36 ± 1	9 ± 1	-
Septate Pigmented I	*D. ramentacea*	Mycelia	-	-	17 ± 2
Sterile Hyaline I	*D. ramentacea*	Mycelia	-	20 ± 1	-
Sterile Hyaline I	*D. ramentacea*	Media	29 ± 2	-	-
*Botrytis* sp. I	*D. ramentacea*	Mycelia	94 ± 1	-	-
*P. decumbens* I	*D. ramentacea*	Mycelia	36 ± 4	-	98 ± 1
*P. decumbens* I	*D. ramentacea*	Media	-	-	99 ± 1
Septate Pigmented III	*D. ramentacea*	Mycelia	47 ± 4	-	-
*Aureobasidium pullulans* I	*D. ramentacea*	Media	82 ± 4	51 ± 2	13 ± 2
*Trametes versicolor*	*D. ramentacea*	Mycelia	-	17 ± 2	-
White Fluffy II	*D. ramentacea*	Media	-	-	12 ± 1
*Coniothyrium sp.*	*D. ramentacea*	Mycelia	31 ± 1	-	-
*Coniothyrium sp.*	*D. ramentacea*	Media	25 ± 2	28 ± 2	-
*Botrytis* sp. II	*D. ramentacea*	Mycelia	-	-	14 ± 1
*Botrytis* sp. II	*D. ramentacea*	Media	97 ± 1	-	-
Red Yeast I	*M. stellatus*	Mycelia	31 ± 2	-	-
Septate Pigmented IV	*M. stellatus*	Mycelia	-	16 ± 3	-
Septate Pigmented IV	*M. stellatus*	Media	23 ± 1	-	70 ± 3
*P. decumbens* II	*M. stellatus*	Media	-	-	12 ± 1
Sterile Hyaline V	*P. palmata*	Mycelia	-	11 ± 1	15 ± 4
Sterile Hyaline V	*P. palmata*	Media	-	-	16 ± 1
*Hypoxylon sp.*	*P. palmata*	Mycelia	-	13 ± 3	30 ± 3
*Hypoxylon sp.*	*P. palmata*	Media	-	-	18 ± 2
*P. decumbens* III	*P. palmata*	Mycelia	-	-	16 ± 5
*P. chrysogenum* I	*P. palmata*	Media	58 ± 3	-	-
*Helicomyces* sp.	*P. palmata*	Mycelia	21 ± 1	-	-
*Helicomyces* sp.	*P. palmata*	Media	-	-	21 ± 5
*P. crustosum* II	*P. palmata*	Mycelia	82 ± 5	-	22 ± 3
*P. crustosum* II	*P. palmata*	Media	39 ± 2	-	-
*Botrytis* sp. III	*P. palmata*	Media	92 ± 1	-	-
Sterile Hyaline VI	*P. palmata*	Mycelia	-	-	13 ± 2
Sterile Hyaline VI	*P. palmata*	Media	19 ± 6	-	-
Sterile Hyaline VII	*P. palmata*	Media	90 ± 2	-	24 ± 2
*Aureobasidium pullulans* II	*P. lanosa*	Mycelia	49 ± 1	34 ± 3	-
Sterile Hyaline IX	*P. lanosa*	Media	-	-	40 ± 5
*Botryotinia fuckeliana*	*P. lanosa*	Mycelia	96 ± 1	-	30 ± 2
*Botryotinia fuckeliana*	*P. lanosa*	Media	41 ± 2	-	-
Sterile Hyaline X	*P. lanosa*	Mycelia	-	-	20 ± 11
Sterile Hyaline XI	*P. purpurea*	Mycelia	22 ± 1	-	-
Sterile Hyaline XI	*P. purpurea*	Media	34 ± 9	-	-
Sterile Hyaline XII	*P. purpurea*	Mycelia	50 ± 1	-	-
Sterile Hyaline XIII	*P. umbilicalis*	Media	-	-	33 ± 1
Sterile Hyaline XIV	*P. umbilicalis*	Mycelia	-	14 ± 1	27 ± 6
Sterile Hyaline XIV	*P. umbilicalis*	Media	22 ± 5	-	-
*Aspergillus sydowii*	*P. umbilicalis*	Media	51 ± 3	-	-
*Aspergillus* sp. II	*A. nodosum*	Mycelia	66 ± 4	-	-
*Aspergillus* sp. II	*A. nodosum*	Media	85 ± 1	-	15 ± 3
Black Hyaline I	*F. spiralis*	Media	41 ± 2	-	-
Septate Pigmented V	*F. spiralis*	Mycelia	39 ± 3	8 ± 1	-
Septate Pigmented V	*F. spiralis*	Media	27 ± 3	-	-
Pigmented Hyaline I	*F. spiralis*	Mycelia	53 ± 4	-	-
White Hyaline IV	*S. latissima*	Media	-	-	30 ± 1
Black Hyaline II	*S. latissima*	Mycelia	-	-	13 ± 5
*P. chrysogenum* II	*S. latissima*	Mycelia	25 ± 4	-	-
*P. chrysogenum* II	*S. latissima*	Media	31 ± 4	-	-
*Aspergillus* sp. III	*S. latissima*	Mycelia	25 ± 1	-	-
Sterile Beige II	*S. latissima*	Media	-	-	14 ± 3
White Hyaline II	*S. latissima*	Mycelia	28 ± 3	-	27 ± 5
White Hyaline II	*S. latissima*	Media	21 ± 1	-	18 ± 9
Pigmented Hyaline IV(Red)	*S. latissima*	Mycelia	-	12 ± 1	-
Pigmented Hyaline IV(Red)	*S. latissima*	Media	-	-	17 ± 1
Sterile Hyaline XVII	*S. arcta*	Mycelia	-	-	13 ± 1
Pigmented Hyaline VII	*S. arcta*	Media	-	15 ± 3	-
White Hyaline III	*S. arcta*	Mycelia	27 ± 7	-	-
White Hyaline III	*S. arcta*	Media	-	-	13 ± 3
*Penicillium spinulosum*	*S. arcta*	Mycelia	65 ± 4	-	-
*Penicillium spinulosum*	*S. arcta*	Media	37 ± 1	-	-
Pigmented Hyaline V	*S. arcta*	Media	87 ± 3	-	82 ± 1
Septate Pigmented VIII	*S. arcta*	Mycelia	-	17 ± 1	-
Septate Pigmented VIII	*S. arcta*	Media	36 ± 2	-	-
*Penicillium soppii* II	*S. arcta*	Media	-	9 ± 3	-
Sterile Hyaline XIX	*U. intestinalis*	Media	-	9 ± 2	22 ± 2
Septate Pigmented VI	*U. intestinalis*	Mycelia	-	-	13 ± 2
Septate Pigmented VII	*U. lactuca*	Mycelia	-	-	18 ± 2
Sterile Hyaline XX	*U. lactuca*	Mycelia	-	24 ± 3	-

^1^ Crude extracts were tested at 200.0 μg·mL^−1^; activity is defined as microbial inhibition significantly differing from negative control; ^2^ Percentage inhibition is represented as the mean of 3 readings ± SE; ^3^ SA: *Staphylococcus aureus*; PA: *Pseudomonas aeruginosa*; CA: *Candida albicans*.

## 4. Conclusions

Our research has demonstrated that marine macroalgae from the Bay of Fundy, Canada, have the potential to be an excellent source of endophytic fungi. Seventy-eight distinct isolates were obtained from 14 algal hosts, with most of the endophytes that could be taxonomically identified belonging to the genera *Penicillium* and *Aspergillus.* The results from the antimicrobial screening on the mycelium and broth extracts of the endophytic fungi suggest that they are a promising source of antimicrobial extracts, with 18 extracts exhibiting >50% inhibition in the screening assays. These crude extracts should be subjected to bioassay-guided fractionation in an attempt to identify the bioactive constituents of the extracts. Further work should also be focused on improving the rate of identification either through the use of molecular techniques, as only 26% of the obtained isolates were identified through this method, or through the induction of conidia or other distinguishing morphological characteristics. The results from this study has led to further work into the endophytic fungi of Atlantic coast marine macroalgae, with a current investigation now focused on the isolation, identification and antimicrobial screening of endophytic fungi from the diverse range of macroalgae present in the Bay of Fundy.
